# Post-Migration Dietary and Lifestyle Transitions and Chronic Disease Risk Among African Migrants in Australia: A Case of Nigerian Migrants

**DOI:** 10.3390/nu18091327

**Published:** 2026-04-22

**Authors:** Kingsley Arua Kalu, Muideen Olaiya, Nse Odunaiya, Blessing Jaka Akombi-Inyang

**Affiliations:** 1 Food Science & Nutrition, School of Science, Translational Health Research Institute, Western Sydney University, Richmond, NSW 2753, Australia; 2 Department of Medicine, School of Clinical Sciences, Monash University, Clayton, VIC 3800, Australia; muideen.olaiya@monash.edu; 3 University College Hospital, University of Ibadan, Queen Elizabeth Road, Ibadan 200285, Oyo State, Nigeria; nodunaiya@com.ui.edu.ng; 4 School of Population Health, Faculty of Medicine and Health, University of New South Wales, Sydney, NSW 2052, Australia; b.akombi@unsw.edu.au

**Keywords:** African, acculturation, nutrition, cardiometabolic risk, dietary intake, food consumption, resettlement, behavioural pattern

## Abstract

**Background:** Migration from low- and middle-income to high-income settings is often accompanied by dietary and lifestyle changes that may increase long-term risk of non-communicable diseases. African migrants represent a growing but under-studied population in Australia, with limited evidence on post-migration nutrition transitions and associated chronic disease risk. This study examined changes in diet and lifestyle among Nigerian-born adults before and after migration to Australia and explored any association with chronic diseases. **Methods:** A pilot cross-sectional study was conducted among adults who migrated from Nigeria to New South Wales, Australia, between 1992 and 2019. Data were collected via a culturally adapted, self-administered online questionnaire assessing socio-demographic characteristics, dietary intake, lifestyle behaviours, and self-reported chronic conditions in the 12 months immediately before and after migration. Descriptive statistics (frequencies and proportions) and inferential analyses (Chi-square tests, McNemar test, and the Bowker test) were used to compare pre- and post-migration behaviours and examine associations with chronic disease outcomes. **Results:** Ninety-three participants completed the survey (mean age 37.0 ± 7.2 years; 50.5% male). Post-migration, regular breakfast consumption declined (−24.3%), while irregular eating (low and moderate) patterns increased (+7.6% and +16.7%). Regular vegetable intake improved (+5.4%), whereas fruit intake remained low (13.0%). Regular consumption of Nigerian local foods decreased markedly (−53.7%), while regular intake of meat (+18.5%), dairy foods, fats (+14.3%), and non-alcoholic beverages increased (+22.8%). Salt use shifted away from the highest-risk category (−22.2%), and smoking and alcohol consumption remained low and stable. Self-reported chronic conditions were uncommon; hypertension (6.5%) and obesity (5.4%) were the most frequently reported. **Conclusions:** Nigerian migrants in Australia experience substantial post-migration dietary and lifestyle transitions that may elevate long-term chronic disease risk despite a currently low reported disease burden. Early, culturally responsive nutrition and lifestyle interventions are needed to support healthy adaptation and prevent the progression of cardiometabolic conditions in this growing migrant population.

## 1. Introduction

Migration from low- and middle-income countries (LMICs) to high-income countries (HICs) has increased substantially over the past several decades, driven by factors such as education, employment, family reunification, and humanitarian pathways [1]. While migration often offers improved economic opportunities and access to healthcare, it is frequently accompanied by profound changes in diet, physical activity, and broader lifestyle behaviours [2,3]. Settlement in high-income food environments is often associated with increased availability, affordability and consumption of energy-dense foods, animal products, sugar-sweetened beverages, and processed foods, alongside reduced physical activity. These post-migration transitions, commonly described as dietary and lifestyle acculturation, have been widely associated with major non-communicable diseases (NCDs, e.g., obesity, hypertension, type 2 diabetes mellitus (T2DM), cardiovascular disease) [2,3], even among migrants who arrive with relatively good health profiles.

Africans, including those from Nigeria, are one of the fastest-growing migrant populations in Australia [4,5,6]. Despite this growth, evidence on their post-migration nutrition and lifestyle patterns remains limited, particularly in comparison with migrant groups from Asia or Europe.

Emerging international evidence suggests that African migrants often experience a nutrition transition characterised by reduced consumption of traditional foods, irregular meal patterns, increased intake of fats and sugars, and greater exposure to sedentary lifestyles [7,8]. However, much of the existing literature relies on qualitative studies and focuses primarily on migrants in Europe or North America. In Australia, data on African migrants’ dietary changes and associated chronic disease risks are sparse. This lack of disaggregated evidence limits the ability of health systems to design culturally responsive prevention strategies tailored to this population [8,9,10].

Understanding pre- and post-migration changes in diet and lifestyle is essential for identifying modifiable risk factors and informing early, culturally appropriate interventions. The period following migration represents a critical window during which health behaviours are reshaped by new food environments, work patterns, social networks, and economic constraints. Without targeted support, these transitions may accelerate the development of chronic diseases that place long-term strain on individuals, families, and health systems.

In this study, we aimed to evaluate post-migration shifts in diet and lifestyle behaviours among Nigerian-born adults residing in New South Wales (NSW), Australia, and to explore their association with self-reported chronic disease risk. We compared nutrition and lifestyle behaviours in the 12 months before migration with those after settlement in Australia. Using Nigerian migrants as a case study, this study seeks to contribute to the limited evidence base on African migrant health in Australia and to inform culturally responsive public health strategies aimed at reducing long-term cardiometabolic risk among African migrant populations.

## 2. Method

### 2.1. Study Design, Participants and Setting

This was a cross-sectional pilot study of Nigerian-born adults (aged ≥ 18 years) who migrated to the state of NSW, Australia. NSW is Australia’s most populous and culturally diverse state and serves as the principal settlement destination for African migrants, including Nigerians, receiving the largest share of the Nigerian-born population nationwide [11]. Recruitment was undertaken from October 2023 to March 2024 through community networks, social media platforms, community leaders and diaspora organisations, including the Nigerian Community–Newcastle and Hunter Region (NCNHR), Hunter African Community Council (HACC). The study employed a within-subject pre–post design. Using participants as their own controls reduces between-individual variability and allows for a more direct assessment of changes in behaviours over time.

### 2.2. Data Collection

An online structured self-administered questionnaire was developed using Qualtrics, comprising three key sections: (i) socio-demographic characteristics, including age, sex, year of migration, marital status, and income bracket; (ii) nutrition and lifestyle behaviours within 12 months immediately before and after migration (to reduce telescoping), covering broad food group consumption patterns-level changes (fruits, vegetables, dairy, grains, meats, seafood, fats, and alcohol) rather than precise portion sizes with validation evidence that directional changes are more reliably recalled than absolute intakes over long intervals [12,13,14,15,16] and physical activity levels (Appendix A Table A1); and (iii) self-reported chronic conditions, including hypertension, diabetes, and obesity. The questionnaire was informed by existing validated tools and adapted through expert consultation to reflect the cultural and dietary nuances of Nigerian migrants and ensure relevance and sensitivity to the Nigerian migrant context. It underwent pre-testing with a small sample of Nigerian community members to ensure clarity, appropriateness, and face validity.

### 2.3. Ethical Considerations

The study was approved by the University of New South Wales Human Research Ethics Committee (HREC Approval No. HC200611), 15 September 2020. Participants provided electronic informed consent prior to completing the questionnaire. Participation was voluntary, and all data were collected in accordance with the Declaration of Helsinki and the National Statement on Ethical Conduct in Human Research [17]. All responses were anonymised and stored securely in accordance with UNSW Human Research Ethics Committee (HREC) protocols.

### 2.4. Data Analysis

Socio-demographic variables and dietary/lifestyle behaviours pre- and post-migration were summarised as frequencies and percentages for categorical variables and mean for continuous variables. Chi-square, Fisher-exact, McNemar, or Bowker tests, were used to compare pre- and post-migration nutrition and lifestyle. Dietary intake frequency was re-categorised into low intake (none or <1 serve/day), moderate intake (1–2 serves/day), high intake (≥3 serve/day), consistent with WHO dietary guidelines [18,19], the Australian Dietary Guidelines [20], and global nutritional risk classification frameworks [10,21,22]. The frequency of consumption of Nigerian local foods was re-categorised into low (never or 1–3 times/month), moderate (2–3 times/week), and high (4–6 times/week or daily), following the food frequency classification used by Ogunba and Akinyele [23]. A complete-case analysis was undertaken for missing variables and no imputation was performed. To evaluate the robustness of the primary results, a sensitivity analysis was conducted in which all analyses were repeated after excluding participants with more than 20 years since migration (Appendix A Table A2, Table A3, Table A4, Table A5, Table A6, Table A7, Table A8, Table A9, Table A10, Table A11, Table A12 and Table A13). This allowed assessment of whether the observed associations were dependent on the inclusion of this long-term migrant subgroup. Full results are provided in Appendix A Table A2, Table A3, Table A4, Table A5, Table A6, Table A7, Table A8, Table A9, Table A10, Table A11, Table A12 and Table A13. Analyses were not adjusted for covariates given the pre–post study design. Statistical significance (2-sided) was set at *p* ≤ 0.05. Data were analysed using STATA version 16.

## 3. Results

### 3.1. Socio-Demographic Characteristics

A total of 93 participants completed the survey (mean age 37.0 ± 7.2 years; 50.5% male). Most (65.6%) arrived in Australia between 2013 and 2019, while the remaining 34.4% migrated between 1992 and 2012 (Table 1). Also, 83% of participants were within ≤10 years post-migration. Most participants were married (67.7%) and within the middle-income bracket (57.1%). Almost all participants (96.7%) reported that they completed a college/University degree.

### 3.2. Pre- and Post-Migration Comparison

#### 3.2.1. Breakfast Consumption

Prior to migration, most participants (76.9%, n = 60) reported regular daily breakfast consumption, while only 10.3% (n = 8) and 12.8% (n = 10) reported low-to-moderate breakfast consumption (Table 2). After migration, regular breakfast consumption declined by 52.6% (n = 41), while about 17.9% (n = 14) and 29.5% (n = 23) reported low and moderate intake post-migration, a slight increase from pre-migration, respectively. This indicates a shift towards a more irregular eating pattern.

#### 3.2.2. Frequency of Salt Use

Table 3 shows a notable shift in salt-use behaviour following migration. The proportion of participants exhibiting ideal behaviour (never adding salt) increased slightly from 1.1% pre-migration to 3.3% post-migration. In contrast, the proportion of participants with low discretionary use (1–2 days/week) or moderate use (3–4 days/week) rose from 4.4% to 10.0% and 12.2% to 22.2%, respectively. Similarly, high frequency use (5–6 days/week) increased from 8.8% to 12.9%, indicating that some individuals reduced daily salt use but did not eliminate it entirely. Importantly, the highest-risk group (everyday salt use) decreased substantially from 73.3% to 51.1%, reflecting a significant improvement in reducing extreme salt consumption. Overall, these findings suggest a positive trend toward lower salt use post-migration, though most participants transitioned to moderate or occasional use rather than achieving ideal behaviour, highlighting the need for continued interventions to promote optimal salt-reduction practices.

#### 3.2.3. Fruits and Vegetables Intake

Fruit and vegetable consumption indicated a clear shift following migration, with associated changes in both the frequency and the proportion of participants meeting the recommended daily serves. Before migration, most participants reported low fruit intake (89.1%, n = 82) compared with those who consumed fruits regularly (high), 10.9% (n = 10). Post-migration data indicated a slight decrease in the low-intake group, while the high-intake group increased slightly as well (Table 4). A similar pattern was observed for vegetable consumption: low intake decreased, while moderate-to-high intake increased significantly post-migration (Table 4).

#### 3.2.4. Consumption of Nigerian Local Foods

Patterns of Nigerian local food consumption showed distinct variation across the sample, with clear differences in intake frequency. A substantial proportion of participants reported a decrease (−53.7%) in consumption of Nigerian local foods post-migration (Table 5).

#### 3.2.5. Grain-Based Foods Intake

Grain-based food consumption showed variation among participants following migration. Participants reported a slight, non-significant increase in pre- and post-migration consumption of grain-based foods, particularly among those with moderate to high intake (Table 6).

#### 3.2.6. Dairy Foods and Fats Intake

Intake of dairy foods and fats increased significantly after migration, including moderate intake (27.5% vs. 45.1%) and high intake (18.7% vs. 33.0%), as shown in Table 7.

#### 3.2.7. Meat Consumption

Low intake decreased markedly, from 16.3% (n = 15) before migration to 5.4% (n = 5) after migration. Moderate intake also declined, from 31.5% (n = 29) pre-migration to 23.9% (n = 22) post-migration. High (regular) intake increased significantly from 52.2% (n = 48) to 70.7% (n = 65) post-migration (Table 8). This indicates a clear shift toward higher meat consumption following migration, with reductions in both low- and moderate-intake categories and a notable increase in regular meat consumption.

#### 3.2.8. Fish and Seafoods Intake

Low intake increased slightly, rising from 36.6% (n = 34) pre-migration to 37.6% (n = 35) post-migration. Moderate intake remained unchanged at 40.9% (n = 38) across both time points. High (regular) intake decreased marginally, from 21.5% (n = 20) to 20.4% (n = 19) post-migration (Table 9). This reflects a largely stable pattern of fish and seafood consumption following migration, with minor shifts away from high (regular) intake toward low intake, and no net change in moderate intake.

#### 3.2.9. Beverage/Non-Alcoholic Intake

Low intake decreased slightly from 20.4% (n = 19) to 10.8% (n = 10) pre-migration. Moderate intake also decreased significantly from 43.0% (n = 40) to 28.0% (30.1) pre- to post-migration. However, participants reported increased regular beverage intake from pre-migration (35.5%, n = 33) to post-migration (54.1%, n = 54) as shown in Table 10.

The sensitivity analysis, which re-ran all statistical tests after removing participants with more than 20 years since migration, demonstrated that patterns of association, significance levels, and pre-/post-migration shifts were largely consistent with the primary results. Although small fluctuations in cell counts and *p*-values occurred due to reduced sample size, these did not change the overall conclusions. Detailed sensitivity-analysis outputs are provided in Appendix A Table A2, Table A3, Table A4, Table A5, Table A6, Table A7, Table A8, Table A9, Table A10, Table A11, Table A12 and Table A13.

#### 3.2.10. Alcohol Consumption

Low intake (never or monthly, 1–3 times/month) was the most common pattern and increased slightly, from 84.3% (n = 59) pre-migration to 88.6% (n = 62) post-migration. Moderate intake (weekly, 2–3 times/week) remained relatively stable, with a minor decrease from 12.9% (n = 9) to 8.6% (n = 6) post-migration (Table 11). High (regular) intake (frequent weekly, 4–6 times/week, or daily) was uncommon and decreased slightly, from 2.9% (n = 2) to 2.2% (n = 2) post-migration.

#### 3.2.11. Smoking of Tobacco Products

Table 12 indicates changes in smoking of tobacco products across three categories: non-smoker/user, occasional, and regular, from pre- to post-migration. Non-smoker/user reported no change post-migration. Also, Occasional use remained unchanged at 1.1% (n = 1) across both time points. Regular use also showed no change, remaining at 1.1% (n = 1) pre- and post-migration. Overall, these findings indicate that smoking prevalence was very low in this population and remained stable after migration.

#### 3.2.12. Physical Activity

Pre-migration physical activity data were available for 63 participants, of whom 36.5% reported being inactive and 63.5% reported engaging in regular physical activity. Post-migration physical activity data were available for 49 participants, with 22.4% reporting inactive and 77.6% reporting regular physical activity (Appendix A). However, data completeness was limited and insufficient responses were reported from all study participants.

#### 3.2.13. Lifestyle-Related Chronic Disease

Table 13 shows the distribution of self-reported conditions in the sample (n = 93) and the proportion among participants who reported at least one condition (condition-positive, n = 10). Hypertension was the most frequently reported condition, affecting 6.5% (n = 6) of all participants and 60% of the condition-positive subgroup. Obesity was the second-most common, present in 5.4% (n = 5) overall and 50% of condition-positive participants. Dental diseases were reported by 2.2% (n = 2) overall and 20% of the condition-positive subgroup. Cancer, osteoporosis, and gout were each reported by 1.1% (n = 1) of the total sample and by 10% of condition-positive participants.

## 4. Discussion

Using Nigerian migrants as a case study, we provide novel/exploratory insights into post-migration dietary and lifestyle transitions among African migrants in Australia. Overall, the findings demonstrate substantial shifts in eating patterns, food choices, and selected lifestyle behaviours following migration, alongside a relatively low but notable self-reported burden of lifestyle-related chronic conditions. These patterns reflect a complex process of dietary acculturation shaped by food availability, affordability, cultural continuity, and the broader Australian food environment. Many studies/reviews have examined the accuracy of recalling past dietary patterns over extended periods of time, providing insight into the reliability of retrospective dietary assessment [12,13,14,15,16]. Although short-term recalls (≤10 years) are generally the most reliable window for reconstructing past diet, studies have demonstrated that recall beyond 20 years can still yield usable and interpretable information about broad dietary patterns rather than precise intake levels [12,13,14,15,16]. In this present study, 83% of participants fell within the short-term recall range (≤10 years; Table 1), while 11% and 4.3% had migrated 11–20 years and 20 years, respectively. Although the proportion of long-interval recall participants (>20 years) was small (n = 4.3%) in this present study, prior research provides reassuring evidence that such data can remain informative. Studies evaluating long-term dietary recall over ≥10 years report modest to moderate agreement for major food groups—especially foods that are stable across the life course [12,13,14,15,16]. Research also indicates that adults recall directional changes in diet (e.g., more or less than before a major life event) more reliably than exact intakes [14,16]. The sensitivity analysis confirmed that the findings were consistent after excluding long-term migrants (>20 years) (Appendix A Table A1, Table A2, Table A3, Table A4, Table A5, Table A6, Table A7, Table A8, Table A9, Table A10, Table A11, Table A12 and Table A13). Also, the study sample was predominantly composed of highly educated participants; this skew may have influenced reported behaviours. It is therefore important to interpret the findings with caution, as the results may not fully reflect the experiences or behaviours of migrants with lower educational attainment.

This aligns with our analytical focus on broad pre-migration dietary patterns rather than precise quantities, thereby reducing susceptibility to long-term recall error. A key finding was the marked decline in regular breakfast consumption following migration, accompanied by increased low and moderate intake. This shift towards irregular eating patterns aligns with broader evidence on dietary acculturation among migrants adapting to time constraints, work schedules, and convenience-driven food environments in high-income countries [3,7,24]. Extensive epidemiological evidence links breakfast skipping with adverse cardiometabolic outcomes, including insulin resistance, obesity, and metabolic syndrome [25]. The observed decline in regular breakfast intake may therefore represent an early behavioural risk factor for future cardiometabolic disease among Nigerian migrants, particularly if sustained over time.

In this study, we report on low salt-use improved post-migration, with a substantial reduction in the highest-risk category (daily discretionary salt use). This redistribution toward lower-frequency salt use may reflect exposure to Australian public health messaging, food labelling, and reformulation policies [26]. However, most participants did not achieve ideal salt-use behaviour, suggesting partial rather than complete behaviour change. Given the well-established association between high salt intake, hypertension, and stroke conditions disproportionately affecting populations of African ancestry [27], these findings highlight both progress and remaining opportunities for targeted salt-reduction interventions tailored to African migrant communities.

Fruit and vegetable consumption patterns showed divergent trends. Fruit intake remained persistently low, with only marginal post-migration improvement, whereas vegetable intake improved substantially, with large reductions in low intake and notable increases in moderate and regular consumption. Similar patterns have been documented among Sub-Saharan African migrants, where vegetables are more easily incorporated into familiar cooking practices, while fruit consumption is constrained by cost, availability of preferred varieties, and perceived taste differences in the host country [3,28]. These findings underscore the need for culturally responsive strategies that specifically address barriers to fruit consumption, including affordability, access, and culturally relevant nutrition education.

This study reported a decline in the regular consumption of Nigerian local foods following migration. While most participants continued to consume traditional foods at least moderately, the shift from regular to moderate intake suggests increasing reliance on host-country foods, ingredient substitution, and modified cooking practices. This pattern mirrors evidence from African migrant populations globally, where traditional food consumption decreases over time due to cost, availability, time constraints, and acculturation pressures [2,29]. In addition, variations in access to culturally specific food products within the destination environment may also contribute to changes in traditional food consumption. Although participants in this study resided within New South Wales, where ethnic food retailers are generally available in major metropolitan areas, differences in proximity to African grocery stores, availability of imported ingredients, and affordability of traditional food products may influence the frequency with which migrants are able to prepare and consume Nigerian local foods. Though moderate retention of traditional foods may support cultural identity and dietary quality, reduced frequency may also alter micronutrient intake and increase reliance on processed alternatives [30,31]. Supporting access to culturally familiar foods and nutritionally equivalent substitutes is therefore critical for preserving dietary quality during settlement.

Beyond availability and affordability, social integration within the host society may also influence dietary choices among migrants. Evidence suggests that migrants’ eating patterns often shift toward those of the host country as individuals become increasingly embedded in new social, occupational, and institutional environments [7,31]. Participation in workplace settings, university campuses, or broader community networks may expose migrants to Australian food norms through shared meals, workplace cafeterias, takeaway foods, and time-constrained eating schedules. Conversely, strong ties within ethnic community networks may facilitate continued consumption of traditional foods through home cooking, community gatherings, and access to culturally specific food markets. Studies among African migrants have shown that dietary behaviours are shaped not only by food availability but also by social networks, cultural identity, and everyday eating environments [31]. Although this study did not directly assess levels of social integration or the contexts in which meals were consumed (e.g., workplace canteens, restaurants, or home-prepared meals), these factors likely contribute to the dietary shifts observed in this population. Future research should therefore examine how social integration, eating environments, and meal contexts influence dietary acculturation and nutrition-related health outcomes among African migrant populations in Australia. Post-migration increases in dairy foods, fats, and meat consumption were pronounced. These shifts likely reflect adaptation to the Australian food environment, where animal products and dairy are more readily available and culturally normative [2,3,7]. While increased dairy intake may confer benefits for calcium and protein adequacy, concurrent increases in fat and meat particularly if dominated by saturated fats and processed meats may elevate long-term cardiometabolic risk [32,33]. These findings are consistent with global evidence indicating that dietary acculturation among African migrants often involves a transition toward energy-dense, animal-based diets [2,32]. Public health interventions should therefore emphasise balanced dietary adaptation, including lean protein sources, portion control, and plant-based alternatives aligned with cultural preferences.

In this study, fish and seafood intake remained largely unchanged following migration, suggesting that this food group may be less sensitive to acculturation than others. Cost, limited availability of preferred species, and unfamiliarity with local varieties have been cited as barriers to increased seafood consumption among African migrants in Australia [34]. Given the cardioprotective benefits of fish consumption, culturally appropriate strategies to promote affordable and acceptable seafood options may offer additional health gains.

The substantial increase in regular consumption of non-alcoholic beverages—likely reflecting sugar-sweetened beverages and fruit juices—is concerning. Strong evidence links sugar-sweetened beverage intake with obesity, type 2 diabetes, hypertension, and cardiovascular disease [33,35,36]. This finding highlights an important target for intervention, particularly as beverage choices are highly responsive to price, marketing, and convenience [37]. Conversely, alcohol consumption remained low and stable, and smoking prevalence was consistently minimal, likely reflecting strong cultural norms and Australia’s comprehensive tobacco control policies [38,39,40]. These protective patterns should be reinforced through culturally responsive health promotion.

Economic factors may also play an important role in shaping post-migration dietary behaviours. Migration to high-income countries is often accompanied by changes in employment opportunities, income levels, and household purchasing power, which may influence food purchasing decisions and dietary patterns. In this study, most participants reported being employed and within the middle-income bracket, suggesting that increased economic stability following migration may have facilitated greater access to a wider range of foods available in the Australian food environment. However, food affordability remains an important determinant of dietary choices, particularly among migrant populations navigating new food systems and cost structures [29]. Previous research has shown that economic constraints can influence the balance between traditional foods and host-country foods, often leading to substitutions based on price, accessibility, and convenience rather than nutritional value [2,3]. Although this study did not directly compare household income before and after migration, changes in economic circumstances may partially explain the increased consumption of certain food groups observed in this study. Future research should therefore examine the role of income trajectories, food affordability, and employment conditions in shaping dietary adaptation among African migrant populations in Australia.

Despite these behavioural shifts, the prevalence of self-reported chronic conditions in this sample was relatively low, with hypertension and obesity being the most common. These rates were substantially lower than national Australian estimates [41,42,43], consistent with evidence that migrant populations often present with a “healthy migrant effect” in early settlement phases, alongside under-diagnosis and reliance on self-report [44,45,46]. However, the dietary and lifestyle changes observed in this study represent established risk pathways for cardiometabolic disease [8,47,48], suggesting that disease burden may increase with longer duration of residence if preventive measures are not implemented.

Collectively, these findings highlight the dynamic nature of post-migration dietary adaptation among Nigerian migrants in Australia. While some changes, such as reduced salt use and increased vegetable intake, are favourable, others, including increased consumption of meat, fats, and sugar-sweetened beverages, may elevate long-term health risks. The coexistence of protective and adverse behaviours underscores the need for nuanced, culturally grounded interventions that support healthy integration into the host food environment without eroding beneficial traditional practices.

### Limitations

Our findings should be interpreted in the context of the following limitations. First, all health conditions and lifestyle behaviours were self-reported, which may have resulted in under or over reporting of true disease prevalence due to limited prior diagnosis, variable health literacy, recall bias, or reluctance to disclose health information. Specifically, retrospective dietary recall is vulnerable to recall bias, telescoping and social desirability. Validation studies show that while quantitative precision declines with longer intervals, broad dietary patterns, especially culturally familiar staples, can often be recalled with modest accuracy [12,13,14,15,16]. Hence, focusing on directional change provides a defensible inference. Second, the sample size was relatively modest and may not be representative of the broader African migrant population in Australia, particularly given the heterogeneity in cultural backgrounds, migration pathways, and settlement experiences within this group.

Third, our pre–post study design limits the ability to infer causal relationships between migration-related lifestyle changes and chronic disease risk. This study could not adjust for key confounders such as age, duration of residence, socioeconomic status, and lifestyle factors due to dataset constraints. Fourth, the absence of objective clinical measurements or validated screening tools (e.g., anthropometry, biochemical markers) may have reduced the precision of prevalence estimates and limited comparability with population-based surveillance data.

Incomplete responses may have reduced statistical power. Also, physical activity data were incomplete (N = 63 pre-migration; n = 49 post-migration); these findings should be interpreted with caution. Given the pilot nature of the study, data imputation was not undertaken, which may further limit generalisability of findings. Overall, our pre–post study design and lack of objective measures of the presence of chronic conditions or dietary intake may limit the robustness of our findings. Despite these limitations, the study provides valuable preliminary insights into post-migration dietary patterns and lifestyle transition among an under-researched migrant population and highlights the need for a more comprehensive/methodological analytical framework for future, larger-scale longitudinal research incorporating objective health measures.

## 5. Conclusions

This study demonstrates a comparatively low self-reported burden of chronic disease among Nigerian Australian migrants. However, hypertension, obesity, and dental disease are strongly influenced by nutrition and lifestyle factors that may shift unfavourably during settlement. These findings underscore the importance of opportunistic screening, culturally responsive health promotion, and early preventive interventions to support healthy lifestyle transitions and reduce long-term chronic disease risk in migrant communities.

## Figures and Tables

**Table 1:** Socio-demographic characteristics of study participants (N = 93).

Characteristic	n (%)
Age (Years)	37.04 ± 7.2
Gender	
Men	46 (50.5)
Women	45 (49.5)
Year Since Migration (YSM) (1992–2019)	
≤10 Years	78 (83.9)
11–20 Years	11 (11.8)
>20 Years	4 (4.3)
Education	
No formal Education	1 (1.0)
High School Completed	2 (2.15)
College/University Completed	90 (96.7)
Marital Status *	
Married (Currently)	63 (67.7)
Never Married	17 (18.3)
Separated	5 (5.4)
Divorced	7 (7.5)
Widowed	1 (1.1)
Employment Status (Last 12 months) *	
Employed	78 (83.9)
Student	9 (9.7)
Self employed	4 (4.3)
Stay-at-home mum/dad	1 (1.1)
Retired	1 (1.1)
Average Income (Last 12 months) (AUD) *	
(≤$37,000)	17 (18.6)
($37,001–90,000)	52 (57.1)
(≥$90,001)	22 (24.2)

Valid percentages exclude missing values, n = 2 (average income) and 2 (gender). * Variables were re-categorised and recoded based on the Australian Bureau of Statistics classification [1,2].

**Table 2:** Breakfast consumption patterns: 12 months pre-migration vs. post-migration (N = 78).

Breakfast Consumption	Pre-Migration n (%)	Post-Migration n (%)	Change (%)
Low intake	8 (10.3)	14 (17.9)	+7.6
Moderate intake	10 (12.8)	23 (29.5)	+16.7
Regular (High) intake	60 (76.9)	41 (52.6)	−24.3

McNemar–Bowker test: χ^2^ (3 × 3 paired, *df* = 3) = 14.78, *p* = 0.002. Five cells (55.6%) had expected counts <5 (minimum = 1.44).

**Table 3:** Frequency of salt intake, 12 months pre-migration vs. post-migration (N = 90).

Salt Use	Pre-Migration (%)	Post-Migration (%)	Change (%)
Ideal behaviour	1 (1.1)	3 (3.3)	+2.2
Low discretionary use	4 (4.4)	9 (10.0)	+5.6
Moderate use	11 (12.2)	21 (22.2)	+10.0
High frequency use	8 (8.9)	12 (13.3)	+4.4
Highest risk use	66 (73.3)	46 (51.1)	−22.2

The McNemar–Bowker test (5-category paired comparison) (8, N = 90) indicated a significant change in category distribution over time (*p* = 0.013). Twenty cells (80.0%) had an expected count <5 (minimum = 0.03).

**Table 4:** Fruit and vegetable intake, 12 months pre-migration vs. post-migration (N = 92).

Intake Category	Pre-Migration n (%)	Post-Migration n (%)	Change (%)
Fruits Intake ^1^			
Low intake	82 (89.1)	80 (86.9)	−2.2
High intake (Regular)	10 (10.9)	12 (13.0)	+2.1
Vegetable Intake ^2^			
Low intake	76 (82.6)	29 (31.5)	−51.1
Moderate intake	0 (0.0)	42 (45.7)	+45.7
High intake (Regular)	16 (17.4)	21 (22.8)	+5.4

^1^ The McNemar test (2 × 2 paired comparison) (1, N = 92) indicated no significant change in category distribution over time (*p* = 0.774). One cell (25.0%) had an expected count <5 (minimum = 1.30). ^2^ The McNemar–Bowker test could not be computed because the table was not square (*p* must be > 1). However, the Pearson chi-square test (2 *df*, N = 92) indicated a significant association between categories (*p* < 0.001). One cell (16.7%) had an expected count <5 (minimum = 3.65).

**Table 5:** Intake of Nigerian local foods, 12 months pre-migration vs. post-migration (N = 69).

Local Nigerian Foods	Pre-Migration n (%)	Post-Migration n (%)	Change (%)
Low intake	0 (0.0)	1 (1.4)	+1.4
Moderate intake	11 (15.9)	47 (68.1)	+52.2
High (regular) Intake	58 (84.1)	21 (30.4)	−53.7

The McNemar–Bowker test could not be computed because the table was not square (*p* must be >1). However, the Pearson chi-square test (2 *df*, N = 69) indicated a marginally significant association between categories (*p* = 0.047). Three cells (50.0%) had an expected count <5 (minimum = 0.16).

**Table 6:** Grain-based foods Intake, 12 months pre-migration vs. post-migration (N = 92).

Grain-Based Foods	Pre-Migration n (%)	Post-Migration n (%)	Change (%)
Low intake	13 (14.1)	10 (10.9)	−3.2
Moderate intake	36 (39.1)	37 (40.2)	+1.1
High (regular) intake	43 (46.7)	45 (48.9)	+2.2

McNemar–Bowker test (3 × 3 paired): χ^2^ (3, N = 92) = 0.699, *p* = 0.873. Ten cells (62.5%) had expected counts <5 (minimum = 0.01).

**Table 7:** Dairy foods and fats intake, 12 months pre-migration vs. post-migration (N = 91).

Dairy Foods and Fats	Pre-Migration n (%)	Post-Migration n (%)	Change (%)
Low intake	49 (53.8)	20 (22.0)	−31.8
Moderate intake	25 (27.5)	41 (45.1)	+17.6
High (regular) intake	17 (18.7)	30 (33.0)	+14.3

The McNemar–Bowker test (3 × 3 paired) (3, N = 91) indicated a significant change in category distribution over time (*p* < 0.001). One cell (11.1%) had an expected count <5 (minimum = 3.74).

**Table 8:** Meat consumption, 12 months pre-migration vs. post-migration (N = 92).

Meat Consumption	Pre-Migration n (%)	Post-Migration n (%)	Change (%)
Low intake	15 (16.3)	5 (5.4)	−10.9
Moderate intake	29 (31.5)	22 (23.9)	−7.6
High (regular) intake	48 (52.2)	65 (70.7)	+18.5

McNemar–Bowker test (3 × 3 paired): χ^2^ (3, N = 92) = 11.34, *p* = 0.010. Eleven cells (68.8%) had expected counts <5 (minimum = 0.01).

**Table 9:** Fish and seafoods intake, 12 months pre-migration vs. post-migration (N = 92).

Fish and Seafood	Pre-Migration n (%)	Post-Migration n (%)	Change (%)
Low intake	34 (36.6)	35 (37.6)	+1.0
Moderate intake	38 (40.9)	38 (40.9)	No change
High (regular) intake	20 (21.5)	19 (20.4)	−1.1

McNemar–Bowker test (3 × 3 paired): χ^2^ (3, N = 92) = 0.111, *p* = 0.990 (primary). Eight cells (50%) had expected counts <5 (minimum = 0.01).

**Table 10:** Beverage/non-alcoholic consumption, 12 months pre-migration vs. post-migration (N = 92).

Beverage/Non-Alcoholic	Pre-Migration n (%)	Post-Migration n (%)	Change (%)
Low intake	19 (20.7)	10 (10.9)	−9.8
Moderate intake	40 (43.5)	28 (30.4)	−13.1
High (regular) intake	33 (35.9)	54 (58.7)	+22.8

McNemar–Bowker test (3 × 3 paired) (3, N = 92). The McNemar–Bowker test indicated a significant change in category distribution over time (*p* = 0.007). Three cells (33.3%) had expected counts <5 (minimum = 2.07).

**Table 11:** Alcoholic consumption, 12 Months pre-migration vs. post-migration (N = 70).

Alcohol Intake	Pre-Migration n (%)	Post-Migration n (%)	Change (%)
Low intake	59 (84.3)	62 (88.6)	+4.3
Moderate intake	9 (12.9)	6 (8.6)	−4.3
High (regular) intake	2 (2.9)	2 (2.2)	−0.7

The McNemar–Bowker test (3 × 3 paired) (3, N = 70) indicated no significant change in category distribution over time (*p* = 0.241). Six cells (66.7%) had expected counts <5 (minimum = 0.06).

**Table 12:** Tobacco smoking prevalence, 12 months pre-migration vs. post-migration (N = 90).

Smoking Status	Pre-Migration n (%)	Post-Migration n (%)	Change (%)
Non-smoker/user	88 (97.8)	88 (97.8)	No change
Occasional	1 (1.1)	1 (1.1)	No change
Regular	1 (1.1)	1 (1.1)	No change

No significant change in category distribution over time.

**Table 13:** Prevalence of self-reported health conditions among participants (n = 93) and proportion among condition-positive subgroup (n = 10).

Condition	Count	% of All (n = 93)	% Among Condition-Positive (n = 10)
Hypertension	6	6.5	60
Obesity	5	5.4	50
Dental diseases	2	2.2	20
Cancer	1	1.1	10
Osteoporosis	1	1.1	10
Gout	1	1.1	10

## Data Availability

Data supporting these findings are available in the UNSW data archive at: https://www.unsw.edu.au/research/data-archive (accessed on 16 February 2026).

## Appendix A

**Table A1:** Physical activity, 12 months pre-migration (N = 63) vs. post-migration (N = 49).

Physical Activity (PA)	Pre-Migration PA N (%)	Post-Migration PA N (%)
Inactive	23 (36.5)	11 (22.4)
Regular	40 (63.5)	38 (77.6)

Data after excluding >20 years since migration (YSM).

**Table A2:** YSM: (≤10 years, 11–20 years).

YSM	Frequency	%
2009–2019 (≤10 Years)	78	87.6
1999–2008 (11–20 Years)	11	12.4
Total	89	100

**Table A3:** Breakfast consumption patterns: pre-migration vs. post-migration (N = 76).

Breakfast Consumption	Pre-Migration n (%)	Post-Migration n (%)	Change (%)
Low intake	8 (10.5)	13 (17.1)	+6.6
Moderate intake	10 (13.2)	23 (30.3)	+17.1
Regular (High) intake	58 (76.3)	40 (52.6)	−23.7

The McNemar–Bowker test (3 × 3 paired) (3, N = 76) indicated a significant change in category distribution over time (*p* = 0.003). Five cells (55.6%) had an expected count <5 (minimum = 1.37).

**Table A4:** Salt use: pre-migration vs. post-migration (N = 86).

Salt Use	Pre-Migration n (%)	Post-Migration n (%)	Change (%)
Ideal behaviour	1 (1.2)	3 (3.5)	+2.3
Low discretionary use	4 (4.7)	7 (8.1)	+3.4
Moderate use	11 (12.8)	20 (22.1)	+9.3
High frequency use	8 (9.3)	12 (14.0)	+4.7
High-risk use	63 (72.1)	45 (52.3)	−19.8

The McNemar–Bowker test indicated a statistically significant change in salt-use behaviour from pre- to post-migration, χ^2^(8) = 16.27, *p* = 0.039, demonstrating that participants’ salt-use patterns shifted in a non-random manner after migration. Degrees of freedom for the McNemar–Bowker test depend on the number of valid categories in your square table. Hence, some categories had zero counts, so SPSS version 30 automatically drops empty rows/columns.

**Table A5:** Fruit intake: pre-migration vs. post-migration (N = 88).

Fruit Intake Category	Pre-Migration n (%)	Post-Migration n (%)	Change (%)
Low intake	79 (89.8)	78 (88.6)	−1.2
Regular (high) intake	9 (10.2)	10 (11.4)	+1.2

The McNemar test (2 × 2 paired) (1, N = 88) indicated no significant change in category distribution over time (*p* = 0.792). No cells (0.0%) had an expected count <5 (minimum = 7.36).

**Table A6:** Vegetable intake: pre-migration vs. post-migration (N = 88).

Vegetable Intake Category	Pre-Migration n (%)	Post-Migration n (%)	Change (%)
Low intake	73 (83.0)	29 (33.0)	−50.0
Moderate intake	0 (0.0)	41 (46.6)	+46.6
High intake (Regular)	15 (17.0)	18 (20.5)	+3.5

The McNemar–Bowker test (3-category paired comparison) could not be computed because the table was not square (*p* must be >1). However, the Pearson chi-square test (2 *df*, N = 88) indicated a significant association between categories (*p* < 0.001). Two cells (33.3%) had an expected count <5 (minimum = 3.07).

**Table A7:** Intake of Nigerian local foods, 12 months pre-migration vs. post-migration (N = 67).

Local Nigerian Foods	Pre-Migration n (%)	Post-Migration n (%)	Change (%)
Low intake	0 (0.0)	1 (1.5)	+1.5
Moderate intake	11 (16.4)	45 (67.2)	+50.8
High intake (Regular)	56 (83.6)	21 (31.3)	−52.3

The McNemar–Bowker test could not be computed because the pre- and post-variables did not share identical category structures. However, A significant association was found between pre- and post-migration Nigerian-food consumption categories, χ^2^(2) = 6.43, *p* = 0.040, with exact tests confirming this pattern (*p* = 0.021), indicating substantial reductions in high traditional food consumption after migration.

**Table A8:** Grain-based foods Intake, 12 months pre-migration vs. post-migration (N = 88).

Grain-Based Foods	Pre-Migration n (%)	Post-Migration n (%)	Change (%)
Low intake	12 (13.6)	8 (9.1)	−4.5
Moderate intake	34 (38.6)	36 (40.9)	+2.3
High intake (Regular)	42 (47.7)	44 (50.0)	+2.3

The McNemar–Bowker test (3-category paired comparison) (3, N = 88) indicated no significant change in category distribution over time (*p* = 0.630). Four cells (44.4%) had an expected count <5 (minimum = 1.09).

**Table A9:** Dairy foods and fats, 12 months pre-migration vs. post-migration (N = 87).

Dairy Foods and Fats	Pre-Migration n (%)	Post-Migration n (%)	Change (%)
Low intake	47 (54.0)	18 (20.7)	−33.3
Moderate intake	25 (28.7)	40 (46.0)	+17.3
High intake (Regular)	15 (17.2)	29 (33.3)	+16.1

The McNemar–Bowker test (3 × 3 paired) (3, N = 87) indicated a significant change in category distribution over time (*p* < 0.001). One cell (11.1%) had an expected count <5 (minimum = 3.10).

**Table A10:** Meat consumption, 12 months pre-migration vs. post-migration (N = 88).

Meat Consumption	Pre-Migration n (%)	Post-Migration n (%)	Change (%)
Low intake	15 (17.0)	3 (3.4)	−13.6
Moderate intake	28 (31.8)	21 (23.9)	−7.9
High intake (Regular)	45 (51.1)	64 (72.7)	+21.6

The McNemar–Bowker test (3 × 3 paired) (3, N = 88) indicated a significant change in category distribution over time (*p* < 0.001). Four cells (44.4%) had an expected count <5 (minimum = 0.51).

**Table A11:** Fish and seafoods intake, 12 months pre-migration vs. post-migration (N = 88).

Fish and Seafoods Intake	Pre-Migration n (%)	Post-Migration n (%)	Change (%)
Low intake	32 (36.4)	34 (38.6)	+2.2
Moderate intake	36 (40.9)	37 (42.0)	+1.1
High intake (Regular)	20 (22.7)	17 (19.3)	−3.4

The McNemar–Bowker test (3 × 3 paired) (3, N = 88) indicated no significant change in category distribution over time (*p* = 0.904). One cell (11.1%) had an expected count <5 (minimum = 3.86).

**Table A12:** Beverage/non-alcoholic consumption, 12 months pre-migration vs. post-migration (N = 88).

Beverage/Non-Alcoholic Consumption	Pre-Migration n (%)	Post-Migration n (%)	Change (%)
Low intake	19 (21.6)	10 (11.4)	−10.2
Moderate intake	39 (44.3)	27 (30.7)	−13.6
High intake (Regular)	30 (34.1)	51 (58.0)	+23.9

The McNemar–Bowker test (3 × 3 paired) (3, N = 88) indicated a significant change in category distribution over time (*p* = 0.005). Three cells (33.3%) had an expected count <5 (minimum = 2.16).

**Table A13:** Alcoholic consumption, 12 months pre-migration vs. post-migration (N = 88).

Alcoholic Consumption	Pre-Migration n (%)	Post-Migration n (%)	Change (%)
Low intake	56 (83.6)	60 (89.6)	No change
Moderate intake	9 (13.4)	5 (7.5)	−5.9
High intake (Regular)	2 (3.0)	2 (3.0)	+6.0

The McNemar–Bowker test (3 × 3 paired) (3, N = 67) indicated no significant change in category distribution over time (*p* = 0.172). Seven cells (77.8%) had an expected count <5 (minimum = 0.06).
